# Engagement, Persistence, and Gender in Computer Science: Results of a Smartphone ESM Study

**DOI:** 10.3389/fpsyg.2017.00602

**Published:** 2017-04-25

**Authors:** Carolina Milesi, Lara Perez-Felkner, Kevin Brown, Barbara Schneider

**Affiliations:** ^1^Education and Child Development, NORC at the University of ChicagoIL, USA; ^2^Educational Leadership and Policy Studies and Sociology, Florida State UniversityTallahassee, FL, USA; ^3^Academic Research Centers, NORC at the University of ChicagoIL, USA; ^4^College of Education and Department of Sociology, Michigan State UniversityEast Lansing, MI, USA

**Keywords:** computer science, experience sampling method, persistence, engagement, gender differences

## Abstract

While the underrepresentation of women in the fast-growing STEM field of computer science (CS) has been much studied, no consensus exists on the key factors influencing this widening gender gap. Possible suspects include gender differences in aptitude, interest, and academic environment. Our study contributes to this literature by applying student engagement research to study the experiences of college students studying CS, to assess the degree to which differences in men and women's engagement may help account for gender inequity in the field. Specifically, we use the Experience Sampling Method (ESM) to evaluate in real-time the engagement of college students during varied activities and environments. Over the course of a full week in fall semester and a full week in spring semester, 165 students majoring in CS at two Research I universities were “beeped” several times a day via a smartphone app prompting them to fill out a short questionnaire including open-ended and scaled items. These responses were paired with administrative and over 2 years of transcript data provided by their institutions. We used mean comparisons and logistic regression analysis to compare enrollment and persistence patterns among CS men and women. Results suggest that despite the obstacles associated with women's underrepresentation in computer science, women are more likely to continue taking computer science courses when they felt challenged and skilled in their initial computer science classes. We discuss implications for further research.

## Introduction

Given the dramatic growth of the computer and technology industries over the past decades, it should come as no surprise that an estimated 1.24 million new computer occupations are expected to be available by the decade's end (Bureau of Labor Statistics, 2013). This is nearly double the rate of job growth projected across all occupational sectors. The need for computer scientists to fill these new jobs, and the requisite training and coding literacy, has become a matter of national importance. Indeed, a federal “Computer Science for All” initiated by President Obama has proposed $4 billion dollars in funding for enhancements in P-12 computer science education, including the training of students and teachers (U. S. Department of Education Office of Innovation and Improvement, 2016).

However, computer science (CS) has actually *declined* in popularity over the last three decades, from 4.3% of postsecondary majors in 1986 to 2.6% in 2012 (NCES, 2012). Women have experienced this decline far more severely than men (Ceci et al., 2014). Only 0.4% of college-bound high school girls express an interest in pursuing CS compared to 3.0% of males (American Association of University Women, 2010). Correspondingly, women represented only 17.6% of CS majors in 2011, down from a peak of 37.1% in 1984 (NCES, 2012). The employment of women in computer and technology industries has also declined, peaking around 35% in the 1980s. In fact, CS is the only STEM field in which women saw a decline in labor representation during this time period (Census, 2013). Even *Google*, which famously purports merit-based hiring practices, recently revealed that only 17% of its technology division is female (Google, 2014). To better understand this disturbing trend, our research uses the Experience Sampling Method (ESM) to (1) understand differences in men and women students' engagement in CS and (2) demonstrate how these differences affect persistence in the field.

### Literature review

The underrepresentation of women in computer science has been much studied but with little consensus as to its cause. Possible suspects are often the same factors attributed to women's lower rates of persistence in many science, technology, engineering, and mathematics (STEM) fields. These include gender differences in aptitude, retention, interest, classroom environment, and of particular interest to our study, student engagement.

### Persistence in STEM

Based on a nationally representative sample of college students, Chen (2013) estimated that 48 percent of bachelor's degree students entering STEM fields between 2004 and 2009 had left these fields by spring 2009, either switching to a non-STEM field or exiting college before earning a degree or certificate. Problematically, more women than men leave STEM fields by switching to a non-STEM major (32 vs. 26%), whereas more men than women leave STEM fields by dropping out of college (24 vs. 14%). After college, men are retained in computing and related professions at a rate nearly double that of women (57–28%) in the first four first after undergraduate study (Corbett and Hill, 2015).

Broadly speaking, research has linked STEM attrition to a weak academic background (Astin and Astin, 1992; Lent et al., 2000). For example, enrollment in Advancement Placement (AP) classes and in math classes beyond Algebra 2 have been associated with an increased probability of choosing and persisting in STEM fields (Adelman, 2006). Griffith (2010) showed much of the gender difference in persistence to a STEM degree is eliminated once the analysis controls for academic preparation. Students' academic performance during college is also key to STEM persistence (Strenta et al., 1994; Ost, 2010; Rask, 2010). Recent reports on STEM attrition showed that taking lighter credit loads in STEM courses in the first year in college, taking less challenging math courses in the first year, and performing poorly in STEM classes relative to non-STEM classes increases students' chances of switching out of STEM majors (Chen, 2013, 2015). It is important to note that this study found that even after accounting for academic performance, women are still more likely than men to leave STEM (Chen, 2013).

Researchers have also found that persistence is associated with psychosocial factors related to STEM disposition. For example, self-efficacy in mathematics and science has been shown to be related to students' choice of college major (Porter and Umbach, 2006) and is a positive predictor of persistence within the STEM fields (Schunk and Pajares, 2002). National longitudinal data from the Cooperative Institutional Research Program (CIRP) indicates mathematics self-concept has been an important predictor of undergraduate students' STEM aspirations, although its influence among women is waning (Sax et al., 2015). Students' deep interest and engagement in mathematics is also related to STEM degree attainment (Engberg and Wolniak, 2013). These psychosocial factors have also been widely used to understand gender disparities in STEM. Gender differences in subjective orientations toward mathematics, for example, emerge early in life, and tend to increase throughout students' educational career (Eccles, 1994; Perez-Felkner, 2015), which in turn help to explain the different majors male and female college students choose (Perez-Felkner et al., 2012; Wang et al., 2013).

### Persistence in computer science

The literature on gender differences in computer science reflects this broader trend. Nearly two decades ago, a meta-analysis of 82 studies found men had more positive attitudes toward computers than women, including greater positive affect and self-efficacy beliefs surrounding their use of computers (Whitley, 1997). Indeed, beliefs matter. According to a recent AAUW report finding women's persistence can be deterred by stereotype-related threats inhibiting women's sense of belonging and implicit biases held by most men majoring in computing, which associate science as a male domain (Corbett and Hill, 2015).

A now classic study at Carnegie Mellon found more women departed from CS majors than men, using 210 interviews with 97 male and female computer science majors (Margolis et al., 2000; Margolis and Fisher, 2002). The authors explain women tended to report experiencing less “intrinsic interest” and single-minded “passion” for programming, irrespective of their initial interest and ability (Margolis and Fisher, 2002). Before entering the major, women CS students at Carnegie Mellon seemed confident in their talent. In comparison to many of their aspiring “boy wonder” peers who seemed to “dream in code” however, women tended to leave in response to lower confidence in face of the stereotype of a particular type of successful CS student and the illusion that they must not have gotten into the major on their own merit (Margolis et al., 2000). Correspondingly, more recent studies using surveys and interviews have identified social isolation, a perceived lack of experience, and a sense of “not belonging” as key barriers preventing women from persisting in their CS studies (Biggers et al., 2008; Powell, 2008; Barker et al., 2009).

Notably, there is evidence these gendered beliefs are malleable. Cohoon (2001) argued that female CS students were retained at rates comparable to males when the CS department had female faculty, strong mentorship, and institutional support. Cheryan et al. (2009) found a less “chilly,” more gender-inclusive environment in the CS classroom was sufficient to increase females' interest in CS to levels comparable to their male peers. Despite scant quantitative evidence on chilly STEM environments for women (e.g., Pascarella et al., 1997), qualitative data continues to indicate this phenomenon exists (Allan and Madden, 2006). Even in highly selective institutions, women who perceive a “chilly” instructional climate are more likely to leave mathematics and computer science (Strenta et al., 1994).

Building off the “chilly environment” concept, researchers have attributed gender differences in CS to cultural factors such as prevailing gender stereotypes/expectations and the lack of female mentorship. Cheryan et al. (2015) review this literature and provide evidence for how popular stereotypes and typical representations of computer scientists make the field seem inaccessible or inappropriate for females. Similarly, Leslie et al. (2015) examine the role of “brilliance” in various academic disciplines and show that fields where “innate talent” is perceived to be of utmost value—such as the STEM fields—also have the lowest representation of women, perhaps due to prevailing stereotypes assuming that females lack the required innate or natural math and science abilities. Given this lack of female representation, the work of Hoh (2009) and Drury et al. (2011) stress the importance of female role models in encouraging women to major in STEM fields such as engineering and computer science.

### Student engagement

In higher education, student engagement has long been used as a predictor of student success in college—notably, student retention and persistence (e.g., Harper and Quaye, 2008; Kuh et al., 2008). These studies are often associated with institutional-level studies of “student success” and tend to focus on behavioral measures of student engagement associated with the National Study of Student Engagement (NSSE), conducted annually on undergraduate first- and fourth-year students (see Kuh, 2009). In a major report on student-level outcomes commissioned by the National Center for Education Statistics, Kuh et al. (2006) conceptualize student engagement as being demonstrated by student behaviors—specifically, “in educationally effective practices”—and supported by institutional conditions for learning, each of which are influenced by additional external and background factors. Building on the work of other major scholars in higher education (e.g., Pascarella, 1980; Pace, 1982; Astin, 1984), these practices are defined as: (1) faculty-student interaction, (2) peer interaction fostering learning, (3) experiences with diversity, (4) cocurricular activities, and (5) student satisfaction with the institution (Kuh et al., 2006). Using the Multiple Institution Database for Investigating Engineering Longitudinal Development, Ohland et al. (2008) use these and related NSSE measures to capture engagement, finding it to be a critical “precursor” for persistence in engineering—in attracting and retaining students. While these studies reliably capture large-scale self-reports of students' behavior over the past year, there are limitations in the ability of these surveys to measure students' lived experience in college (Porter, 2011).

Few studies have looked beyond these behavioral measures of student engagement to assess its effects on longer-term decisions regarding their college majors, careers, and future goals (Harackiewicz et al., 2000; Shernoff and Hoogstra, 2001; Gasiewski et al., 2012). Even fewer have examined the classroom engagement of college students (Mills and Fullagar, 2008; Steele and Fullagar, 2009; Asakawa, 2010). Gasiewski et al. (2012) offered a multidimensional perspective in a rigorous mixed method study of students studying their academic engagement in STEM gateway courses. Nevertheless, the authors' main findings were behaviorally-oriented: authors found academic engagement was positively associated with (1) signals from STEM faculty “gatekeepers” that they were open to students' success (i.e., not being chilly) and (2) proactive student academic behavior (e.g., asking questions, seeking assistance). The studies noted thus far examine how student behaviors and institutional supports affect students' engagement and in some cases longer-term outcomes. They tend not however to assess how students experience engagement in academic and related activities, in particular in relation to their persistence in specific STEM gateway courses.

We build on this literature by providing a more precisely grounded definition of “engagement” with which to examine our longitudinal case study of computer science women at two institutions. Our study is the first to examine student engagement in CS as a function of their level of interest, challenge, and skill with college coursework by using the ESM to collect real-time data on student experiences. Specifically, our study approaches the operationalization of engagement in “flow” theory as a state in which high challenge is matched by high skill (Csikszentmihalyi, 1990). Shernoff et al. (2003) show that levels of engagement in high school vary substantially based on academic conditions, also noting the importance of engagement for keeping students' stimulated and eager to learn. Similar findings with regards to the positive effects of engagement on motivation and learning have been found at all stages of the K-12 education system (Marks, 2000; Rathunde and Csikszentmihalyi, 2005; Schweinle et al., 2006; Shernoff and Schmidt, 2008). Here, we apply this approach to college students taking computer science classes, with the intent of better understanding how differences in men and women's engagement in CS may help account for gender disparities in students' success in the field.

## Methodology

### Data

The data for this study were collected from a sample of students majoring (or interested in majoring) in computer science/engineering at two Research I institutions in the Midwest with highly rated computer science/engineering programs, one private (School A) and one public (School B). Data were collected through three primary instruments: (1) a real-time survey, based on the ESM, prompted and administered through smartphones on Fall 2013 and Spring 2014; (2) a pre-survey distributed to students in Fall 2013 prior to their receiving the smartphones aimed at obtaining students' background information and probing their prior academic and computer-science related experiences; and (3) students' administrative records on their declared majors, courses, grades, and credits accumulated in computer science from Fall 2013 through Fall 2014.

As compared to other methods of observational data, the ESM allows us to evaluate the engagement of students during varied activities and environments throughout the course of the day, all in real-time (Csikszentmihalyi and Larson, 1987; Hektner et al., 2007; Zirkel et al., 2015). ESM acquires information on where, when, and how a student is feeling at that moment. These responses can be aggregated within individuals over time. ESM is often referred to as an Ecological Momentary Assessment (EMA) as it is conducted in a natural setting that is outside of a laboratory condition. Over the course of a week during Fall 2013 and again during Spring 2014, participating students received smartphones programmed with an ESM app that beeped eight times a day, at which point they were prompted to fill out a short questionnaire including open-ended and scaled items (see Appendix in Supplementary Material for full ESQ). The purpose of the ESQ was to obtain information about students' momentary cognitions, affect, and behaviors both in and outside of academic contexts. Each questionnaire asked students to indicate where they were, who they were with, and what they were doing. Additional detail was requested regarding their location and main activity. Students were also asked whether their activity entailed the use of technology as well as a series of 15 questions probing how they felt about the activity on a Likert scale.

Each institution's registrar and admissions office provided information on student's characteristics and their coursetaking behavior from Fall 2013 through Fall 2014.^1^ Administrative data provided information on students' academic year, college courses taken, grades received, cumulative college GPA, high school GPA, SAT, and/or ACT scores, home addresses, high school location, student race/ethnicity, athlete status, and whether the student is a US citizen, first generation college student, or transfer student.

### Outcome variables

This study relies on administrative data to measure students' persistence in computer science in two different ways. The first measures the percentage of credit hours coming from CS classes in Fall 2014, a year after students began participating in this study and one term after students were asked to participate in the ESM data collection. As an example, if a student took 6 credit hours in CS out of the 12 credit hours he completed in Fall 2014, he would be counted as having 0.50 credit hours in CS. The second measure compares the percentage of credit hours in computer science across two terms: Fall 2013 and Fall 2014. Students' persistence in CS was categorized as a decrease, increase, or no change in the percentage of credit hours in CS. Following the example mentioned above, if that same student had taken 3 of the 12 credit hours in CS a year earlier (Fall 2013), he would be counted as having increased his involvement in CS from 0.25 to 0.50 percentage of credit hours in CS. We used CS credit hours rather than CS courses in order to standardize coursetaking measures across schools.

### Covariates and other measures

Responses to the ESM questionnaire were used to measure variations in students' levels of engagement across contexts. Based on the concept of “flow,” we define engagement as the combination of challenge, interest, and skill. Specifically, we rely on student's responses to these three questions:

- Was this activity *challenging* to you?
- Was this activity *interesting* to you?
- How *skilled* are you in this activity?

Every time students were beeped, they reported whether the current activity ranged from “not at all” to “very” (scaled from 1 through 6) challenging, interesting, and skilled. We calculated the average levels of challenge, interest, and skill for each student. In this analysis, we combine students' ESM reports from Fall 2013 and Spring 2014. Because we are particularly interested in students' engagement across contexts, we calculated these averages separately for (i) beeps in which the student reported being involved in a CS academic activity, including CS classes or labs, studying or doing homework related to CS, and activities related to CS such as programming, coding, or learning a CS language; (ii) beeps in which the student reported being involved in a non-CS academic activity, such as classes, labs, homework, group projects, and studying subjects other than CS. We did not include beeps in which the student reported being in a non-academic activity such as socializing or at work.

### Analytic sample

In the Fall of 2013 we recruited a total of 167 students across the two postsecondary institutions involved in this study. We dropped from the analysis students who were no longer enrolled in Fall 2014 either because they had graduated, were suspended, or withdrew from the university (*n* = 27). We also dropped from the analysis students with no eligible beeps either because beeps were responded to outside of the study period or had no information that would allow us to code the main activity (*n* = 2). Finally, we dropped students with eligible beeps but with zero beep responses in either CS-academic activities or non-CS-academic activities (*n* = 19). Our analytic sample is composed of 123 students who had on average 6.7 beeps in CS-academic activities and 26.5 beeps in non-CS-academic activities (“other” academic activities).

Table 1 below shows key background characteristics of students in our analytic sample. About one-third of the sample at both universities was women, who were oversampled given the relative lack of female computer science majors. Over three-fourths of the students were either white or Asian, and just over 12% belonged to an underrepresented racial or ethnic minority group in STEM. Though freshmen account for a slightly larger percentage of students at School B than School A, the overall distribution of students among cohorts looks similar across universities, with about 70% of students being either freshmen or sophomores. Notably, the two schools differ in the proportion of students who have declared a CS-related major, primarily because School A does not require students to declare majors in their freshman and sophomore years. At both universities, just under one-quarter of declared majors chose a discipline that did not include computer science. However, the great majority of these were enrolled in a CS class in Fall 2013.

**Table 1:** **Demographic and academic characteristics of students in analytic sample, by institution**.

	**School A**		**School B**		**Schools A and B combined**	
	***n***	**%**	***n***	**%**	***n***	**%**
**GENDER**						
Male	45	68.18	37	64.91	82	66.67
Female	21	31.82	20	35.09	41	33.33
**RACE/ETHNICITY**						
White	28	42.42	45	78.95	73	59.35
Asian	16	24.24	6	10.53	22	17.89
Underrepresented in STEM^*^	11	16.67	4	7.02	15	12.20
International	5	7.58	1	1.75	6	4.88
Unspecified	6	9.09	1	1.75	7	5.69
**COHORT**						
Freshmen	24	36.36	25	43.86	49	39.84
Sophomore	21	31.82	16	28.07	37	30.08
Junior	21	31.82	16	28.07	37	30.08
**MAJOR IN FALL 2013**						
Computer science^**^	16	24.24	46	80.70	62	50.41
Physical science, engineering	6	9.09	9	15.79	15	12.20
**MATH**						
Other STEM	6	9.09	0	0.00	6	4.88
Non-STEM	2	3.03	2	3.51	36	29.27
Common year/Undeclared	36	54.55	0	0.00	4	3.25
Total	66		57		123	

*Source: School A and School B Registrar*.

* *Includes Hispanic, Black or African American, Hawaiian or Pacific Islander, Native American, and Multiple Races*.

** *In the case of a handful of students who reported multiple majors, the student was counted as a computer science major if one of the majors recorded was computer science related*.

We compared our analytic sample to the national population of students majoring in CS using the Educational Longitudinal Study (National Center for Education Statistics, 2002), a nationally representative, longitudinal study of 10th graders in 2002, followed throughout secondary and postsecondary years. Both ELS and our dataset demonstrate the extent to which White and Asian students continue to pursue CS degrees, while minority students continue to be underrepresented. Because our study aimed to study gender disparities in the field, we oversampled female students and therefore, have a higher representation of women than ELS. Our sample is similar to ELS in students' career attainment values and subjective orientations toward mathematics. An important difference with ELS is the fact that students in our sample have about 15 percent higher SAT match and verbal scores than students majoring in CS as sophomores in most, highly, and very competitive institutions (results available upon request).

### Analytic strategy

The study was designed as a pilot to determine the feasibility of analyzing the association between students' engagement and their persistence in CS. Since we did not find any meaningful differences in student characteristics across schools or any differences in their patterns of engagement, this analysis combines students across institutions. We carried out multivariate analysis for each of the dependent variables specified above. We estimated an OLS regression predicting the percentage of CS credits in Fall 2014, and a multinomial logistic regression predicting the change in CS credits from Fall 2013 to Fall 2014. In this second regression model, the dependent variable had three mutually exclusive categories: “increase in percentage of CS credit hours,” “same percentage of CS credit hours,” and “decrease in percentage of CS credit hours;” we used the latter as the reference category. Because we are particularly interested in how engagement affects female and male students differently, we estimated separate models by gender.

## Results

Table 2 shows mean and standard deviation of the three items that measure student engagement, separately for computer science academic activities and other academic activities. Both male and female students report higher levels of challenge and interest in academic activities related to CS than academic activities related to other subjects. The reverse is true for the third component of engagement: both male and female students report feeling less skilled in CS than in non-CS academic activities. It is important to note that there are no statistically significant differences between men and women in either the reports of challenge, interest, or skill.

**Table 2:** **Student engagement, by context and gender**.

	**Females**		**Males**	
	**Mean**	**Std. Dev**.	**Mean**	**Std. Dev**.
**COMPUTER SCIENCE ACADEMIC ACTIVITIES**				
Was this activity challenging to you?	3.92	1.18	3.92	1.02
Was this activity interesting to you?	3.95	1.14	4.23	1.00
How skilled are you in this activity?	4.21	0.63	4.19	0.85
**ACADEMIC ACTIVITIES IN NON-COMPUTER SCIENCE SUBJECTS**				
Was this activity challenging to you?	3.31	0.58	3.16	0.69
Was this activity interesting to you?	3.72	0.71	3.67	0.68
How skilled are you in this activity?	4.39	0.54	4.50	0.50

*ESM responses on 6-point scale, from “not at all” (1) to “very” (6)*.

Table 3 displays descriptive statistics for the two different measures of persistence in computer science described above. Table 3A shows that, on average, male and female students in our study took about a third of their credit hours in CS in Fall 2014. One in four students did not take any CS credit hours in Fall 2014 and also one in four students took more than half of their credit hours in CS. The distribution of this outcome differs slightly by gender: while half of women took 46 percent of their credit hours in CS, half of male students took 25 percent or more of their credit hours in CS. Table 3B shows that approximately 38 percent of students had the *same* percentage of CS credit hours in Fall 2014 as in Fall 2013; 37 percent of students *increased* the percentage of CS credit hours during this period; and about 24 percent of students *reduced* their CS credit hours 1 year later. Despite the underrepresentation of women in CS, women in our study were equally likely to persist in CS. In fact, throughout the course of the year, women tended to increase their CS coursetaking slightly more than men and to decrease their CS coursetaking slightly less than men.

Table 3**Persistence in Computer Science, by gender**.

**Table:** 

**A. Percentage of credit hours in Computer Science during Fall 2014**.			
	**Females**	**Males**	**Total**
*n*	41	82	123
Mean	0.361	0.343	0.349
Std. Dev.	0.283	0.292	0.288
25th percentile	0.000	0.000	0.000
Median	0.462	0.258	0.286
75th percentile	0.500	0.533	0.500

**Table:** 

**B. Change in percentage of credit hours in Computer Science from Fall 2013 to Fall 2014**.						
	**Females**		**Males**		**Total**	
	***n***	**%**	***n***	**%**	***n***	**%**
No change	31	38	16	39	47	38
Increase	32	39	14	34	46	37
Decrease	19	23	11	27	30	24
Total	82	100	41	100	123	100

Table 4 displays OLS regression estimates predicting the percentage of credit hours in Computer Science. Using the dependent variable described in Table 3A, these estimates stem from two separate models, one for women and one for men. Estimates for men do not show any statistically significant association between engagement and the percentage of credit hours in CS a term after the ESM data collection ended, but there is a positive association between male students' self-reports on how skilled and challenged they feel in CS and their subsequent persistence in CS. Results show that among women, feeling skilled in CS academic activities has a positive and statistically significant association with persistence in CS.

**Table 4:** **OLS regression estimates predicting percentage of credit hours in computer science in Fall 2014, by gender**.

	**Females**			**Males**		
	**Coefficient**	**Std. Err**.	***P* > |t|**	**Coefficient**	**Std. Err**.	***P* > |t|**
**COMPUTER SCIENCE ACADEMIC ACTIVITIES**						
Challenge	0.00	0.06	0.99	0.05	0.04	0.24
Interest	−0.01	0.05	0.85	0.03	0.04	0.42
Skill	0.14	0.08	0.08	−0.02	0.05	0.74
**ACADEMIC ACTIVITIES IN NON-COMPUTER SCIENCE SUBJECTS**						
Challenge	0.02	0.10	0.82	−0.04	0.05	0.47
Interest	0.02	0.06	0.80	−0.03	0.05	0.52
Skill	−0.03	0.09	0.75	0.03	0.06	0.60
Constant	−0.21	0.62	0.73	0.22	0.33	0.52

Table 5 displays estimates from a multinomial logistic regression predicting change in the percentage of credit hours in computer science from Fall 2013—when the ESM study began—to Fall 2014—a term after the ESM data collection ended. These models use the dependent variable described in Table 3B and are estimated separately for women and men. Estimates show that among men, students who report feeling more skilled in CS academic activities are also more likely to increase their percentage of credit hours in CS over the course of a year. Consistent with the OLS regression results, these estimates show that among men, while challenge in CS academic activities is positively associated with an increase in the percentage of credit hours in CS, challenge in other academic activities is negatively associated with an increase in the percentage of credit hours in CS. This pattern of association between engagement and persistence is not observable among women. However, it is important to note that female students who reported feeling more skilled in academic activities related to subjects other than CS are more likely to increase their CS credit hours.

**Table 5:** **Multinomial logistic regression estimates change in percentage of credit hours in computer science Fall 2013-Fall 2014, by gender**.

	**Females**				**Males**			
	**Coeff**.	**Std. err**.	**Odds ratio**	***P* > |z|**	**Coeff**.	**Std. err**.	**Odds ratio**	***P* > |z|**
**INCREASE IN CS CREDIT HOURS VS. NO CHANGE**								
**Computer Science Academic Activities**								
Challenge	−0.13	0.49	0.88	0.79	0.55	0.36	1.73	0.12
Interest	0.05	0.47	1.05	0.92	−0.21	0.32	0.81	0.51
Skill	0.66	0.71	1.93	0.35	0.69	0.42	1.99	0.10
**Academic activities in non-Computer Science Subjects**								
Challenge	1.76	0.94	5.81	0.06	−0.39	0.44	0.67	0.37
Interest	−0.73	0.57	0.48	0.20	−0.65	0.42	0.52	0.12
Skill	0.16	0.80	1.18	0.84	0.70	0.53	2.01	0.19
Constant	−7.81	5.75	0.00	0.17	−3.30	2.73	0.04	0.23
**DECREASE IN CS CREDIT HOURS VS. NO CHANGE**								
**Computer Science Academic Activities**								
Challenge	0.18	0.64	1.19	0.78	−0.04	0.38	0.96	0.91
Interest	0.83	0.63	2.29	0.19	0.00	0.36	1.00	0.99
Skill	−1.43	1.02	0.24	0.16	0.49	0.47	1.63	0.30
**Academic activities in non-Computer Science Subjects**								
Challenge	−0.02	1.01	0.98	0.99	0.91	0.57	2.49	0.11
Interest	−0.91	0.66	0.40	0.17	−0.51	0.49	0.60	0.29
Skill	0.73	1.02	2.08	0.47	0.18	0.62	1.19	0.77
Constant	1.55	5.78	4.69	0.79	−5.04	3.40	0.01	0.14

## Conclusion

This study uses the ESM to determine how male and female students at two different postsecondary institutions experience engagement in academic settings and how that engagement affects their persistence in pursuing a computer science degree. Results are robust across two alternative measures of persistence in computer science: Among women, reporting feeling more skilled in CS and non-CS academic activities is positively associated with persistence in CS. Among men, while challenge in CS academic activities is positively associated with persistence, challenge in non-CS academic activities is negatively associated with persistence in CS. These results are consistent with explanations of the leaky STEM pipeline in college attributed to (1) stereotypes of women as being less skilled than men in math and science and (2) cultural norms that portray computer science as being more “appropriate” for men than women. When faced with challenging CS coursework, male students redouble their efforts while female students who perceive their skills to be lacking tend to drop out. A third possibility is that women who begin college as a CS major tend to find computer science less intrinsically rewarding than men or perhaps fear that their gender will preclude a successful career in CS regardless of their passion for the field.

However, our study was originally conceived as a pilot to demonstrate “proof-of-concept” rather than yield definitive results. In that regard, we were successful at recruiting men and women computer science college students, implementing the ESM data collection, and conducting analysis that links ESM with administrative data on students' coursetaking behavior. Our analysis is limited on three important counts. First, the small sample size does not allow us to conduct more complex analysis that takes into account additional student characteristics or coursetaking behaviors associated with persistence in CS, such as race/ethnicity, first generation status, or coursework and performance during the first year in college. Second, our study does not capture the wide range of postsecondary institutions that offer computer science programs. The institutions included in this study have relatively high selectivity, and the students who attend these institutions have stronger academic backgrounds than their national counterparts. Given that a solid academic background has been linked to CS persistence, it is not surprising that students in our sample exhibit higher rates of persistence in CS than national estimates would suggest. Third, although longitudinal by design, our 1-year data collection period does not allow us to track persistence to degree attainment for earlier cohorts of students or identify students who failed to persist for the juniors in our sample. Expanding this study across different types of institutions and over a longer time period would enable us to conduct further analyses on the relationship between engagement and persistence, ultimately providing insight into the factors that determine why more men than women obtain degrees in computer science.

## Ethics statement

This study was carried out in accordance with the recommendations of the Institutional Review Board (IRB) of NORC at the University of Chicago with written informed consent from all subjects. All subjects gave written informed consent in accordance with the Declaration of Helsinki. The protocol was approved by the IRB of NORC at the University of Chicago.

## Author contributions

All authors contributed extensively to the work presented in this paper. CM conceptualized analysis, managed data collection, carried out analysis, interpreted results, and wrote initial draft. LP conceptualized analysis, reviewed various drafts, and co-wrote final paper. BS conceived and designed overall study, conceptualized theoretical framework, reviewed preliminary and final results, and co-wrote paper. KB conceptualize analysis, co-managed data collection, reviewed results, and co-wrote final paper.

## Funding

This material is based upon work supported by the National Science Foundation under Grant Numbers HRD-1232139 and DRL-0815295. Any opinions, findings, and conclusions or recommendations expressed in this material are those of the authors and do not necessarily reflect the views of the National Science Foundation.

### Conflict of interest statement

The authors declare that the research was conducted in the absence of any commercial or financial relationships that could be construed as a potential conflict of interest.

## Footnotes

^1^This study was conducted in compliance with institutional review board protocols for human subjects research.
