# Supplementary material for: Engagement, Persistence, and Gender in Computer Science: Results of a Smartphone ESM Study
Source: Front Psychol. 2017 Apr 25;8:602. doi: 10.3389/fpsyg.2017.00602 (PMC5403895; doi:10.3389/fpsyg.2017.00602)
Supplement: Supplementary file 1 [file DataSheet1.pdf]

Appendix

ESM Questionnaire

WHEN YOU WERE BEEPED…

1.1. Where were you? (required)

A. In class, Computer Sciences
B. In class, not Computer Sciences
C. On campus, not in class
D. At home
E. In public, not work or school
F. At my job site
G. Other

1.2 Please provide more detail: (required)

____

2.1. Who was the primary person/people you were with? (required)

A. N/A (I was alone)
B. Classmate(s)
C. Friend(s)
D. Family member(s)
E. Significant other
F. Co-worker(s)
G. Faculty/staff
H. Other

3.1. What was the main thing you were doing? (required)

A. Academic work/study
B. Leisure
C. Socializing
D. Personal maintenance (e.g. sleep, eating, personal hygiene)
E. Employment
F. Other activity

3.2. Please provide more detail on what you were doing:

____

4. **Were you doing this because you:**
   A. Wanted to
   B. Had to
   C. Had nothing else to do

5. **Is the main thing you were doing (choose one):**
   A. More like work
   B. More like play
   C. Both
   D. Neither

6. **If you were using technology; what kind of device was it?**
   A. N/A
   B. Computer
   C. Tablet
   D. Smartphone (other than study phone)
   E. Gaming device
   F. Other

7. **How did you feel about your main activity? (6-point scale: not at all/very)**
   7.1. Was this activity challenging to you?
   7.2. Was this activity interesting to you?
   7.3. Was the activity important to you?
   7.4. How skilled are you in this activity?
   7.5. Did you enjoy what you were doing?
   7.6. How well were you concentrating?
   7.7. How important is this activity in relation to your future goals/plans?
   7.8. Were you living up to your own expectations?
   7.9. Were you living up to the expectations of others?
   7.10. Were others expecting a lot from you?
   7.11. Did the situation allow you to be involved or to act?
   7.12. Did you have the abilities to deal with the situation?
   7.13. Were you succeeding at what you were doing?
   7.14. Did you wish you were doing something else?
   7.15. Did you feel good about yourself?

8. **How were you feeling when you were beeped? (6-point scale: not at all/very)**
   8.1 Happy
   8.2 Energetic
   8.3 Anxious
   8.4 Competitive

8.5 Lonely
8.6 Proud
8.7 Cooperative
8.8 Bored
8.9 Self-confident
8.10 Overwhelmed
